# Maternal Treatment with Agonistic Autoantibodies against Type-1 Angiotensin II Receptor in Late Pregnancy Increases Apoptosis of Myocardial Cells and Myocardial Susceptibility to Ischemia-Reperfusion Injury in Offspring Rats

**DOI:** 10.1371/journal.pone.0080709

**Published:** 2013-11-21

**Authors:** Zhu Jin, Wenhui Zhang, Hailiang Yang, Xiaofang Wang, Yanqian Zheng, Qiaoyan Zhang, Jianming Zhi

**Affiliations:** 1 Department of Physiology, Shanghai Jiao Tong University School of Medicine, Shanghai, China; 2 Department of Laboratory, Yuncheng City Center for Disease Control and Prevention, Yuncheng, Shanxi, China; 3 School of Pharmacy, Second Military Medical University, Shanghai, China; Thomas Jefferson University, United States of America

## Abstract

Epidemiological studies have demonstrated that offspring born to mothers preeclampsia (PE) are at increased risk for developing cardiovascular diseases after birth, but the underlying mechanism is unknown. Angiotensin II receptor type 1 autoantibody (AT1-AA), an agonist acting via activation of the AT1 receptor, is believed to be involved in the pathogenesis of both PE and fetal growth restriction. The aim of the present study was to confirm the hypothesis that prenatal AT1-AA exposure increases the heart susceptibility to ischemia/reperfusion injury (IRI) in the offspring in an AT1-AA-induced animal model of PE, and determine whether or not the increase of maternal AT1-AA level is a factor contributing to sustained abnormalities of the heart structure during infancy. The hearts of 45-day-old offspring rats were studied using Langendorff preparation to determine the susceptibility of the heart to IRI. The results showed that the body weight of the maternal rats was not significantly different between the study and control groups, but the body weight of their offspring in AT1-AA group was decreased slightly at day 21 of gestational age, and at day 3 after birth. Although the heart weight index was not significantly affected at all ages examined, AT1-AA significantly increased the size of myocardial cells of the left ventricle (LV) at the age of 45 days. AT1-AA gained access to fetal circulation via the placenta and induced apoptosis of fetal myocardial cells. AT1-AA also significantly delayed recovery from IRI and affected the LV function of 45-day-old offspring. This was associated with a significant increase in IRI-induced LV myocardial infarct size. These results suggest that AT1-AA induced abnormal apoptosis of fetal myocardial cells during the fetal period and increased the cardiac susceptibility to IRI in adult offspring.

## Introduction

Hypertension is a degenerative disease associated with aging, genetic, environmental and behavioral factors, but the etiological mechanism of hypertension has not been fully explained. Evidence from both human and animal studies suggests that the hypertensive manifestations in adulthood are associated with environmental factors during fetal life^1-3^. This phenomenon is known as fetal origins of adult disease. Children born to mothers who smoke during pregnancy are at an increased risk of cardiovascular disease (CVD) [1]. Animal experiments have confirmed that exposure to cadmium [2], or cocaine [3], during pregnancy reprograms cardiovascular development of the offspring, which in turn may conduce to a long-term increased risk of CVD. Low birth weight, childhood growth, and subsequent disease in adulthood have all been linked to several adverse environmental influences during early development [4]. In addition, a number of studies suggest an association between the risk of CVD and abnormal intrauterine growth, despite normal birth weight [5,6].

PE is a frequent complication of pregnancy. Long-term follow up studies have demonstrated that babies born to mothers with PE are more likely to develop CVD, including hypertension and coronary artery disease, in adult life. Further studies have revealed that myocardial cell apoptosis during the fetal period is the major cause of fetal demise and the occurrence of adult CVD [7-10], although the process that mediates these effects is poorly understood. Using a transgenic mouse model that expressed a conditionally active caspase exclusively in the myocardium, they demonstrated that ongoing low levels of myocardial cell apoptosis (23 myocardial cells per 10^5^ nuclei) were sufficient to cause fatal dilated cardiomyopathy [11]. So, excessive myocardial cell apoptosis may cause cardiac abnormalities at both fetal and postnatal stages, but the mechanism underlying fetal rat myocardial cells (FRMCs) apoptosis remains unclear. 

In 1999, Wallukat et al. [12] first reported that the angiotensin II receptor type 1 autoantibody (AT1-AA) is present in patients with PE, but not in healthy pregnant women or those with essential hypertension. Further research found that the target point of AT1-AA is in the second extracelluar loop of AT1 receptor, and AT1-AA, acting as the natural agonist Ang II [13]. AT1-AA may play an important role in promoting the development of PE, possibly through inducing the expression of endothelin, tumor necrosis factor (TNF)-α and soluble fms-like tyrosine kinase-1 (sFlt-1) [14]. Thus, AT1-AA is believed to be an important factor contributing to the development of PE. In our previous study [15,16], we confirmed that AT1-AA induced apoptosis in cultured myocardial cells of neonatal rats in vitro, and promoted adult rat ventricular remodeling in vivo. Irani et al. [17] demonstrated that AT1-AAs may contribute to intrauterine growth restriction (IUGR) through a direct detrimental effect by activating AT1 receptors on multiple fetal organs, and indirectly by inducing small placentas characterized by increased trophoblast apoptosis. 

There is no direct evidence that AT1-AA from pregnant women with PE can pass through the placenta into the fetal circulation to affect fetal development and promoting apoptosis of myocardial cells, thus leading to an increased cardiac susceptibility to ischemic insults in postnatal life. The purpose of the present experimental research was to investigate the apoptosis of myocardial cells in fetal rats induced by AT1-AA, which also exists in the cord blood of women with PE, and study the related signaling pathway. In addition, LV function and susceptibility to IRI was examined in their 45-day-old offspring.

## Materials and Methods

### Animals

SPF Wistar rats (Experimental Animal Center of Shanghai Jiaotong University School of Medicine, Shanghai, China) were fed normal rat chow and tap water *ad libitum* with a 12:12 h light-dark cycle (lights on at 19:00 h) at a constant ambient temperature (23 ± 2°C) and humidity (60% ± 5%). All experimental protocols were approved by the Experimental Animal Care and Use Committee of Shanghai Jiao Tong University School of Medicine.

Virgin female Wistar rats (240~260 g) were mated overnight with sexually experienced males, and pregnancy was confirmed by the presence of a vaginal plug of semen in the mating cages on the following morning (day 1 of pregnancy). The 16 rats were equally randomized into saline control group and AT1-AA treated group. AT1-AA (100 μL PBS, titer >1:640) was administered to the pregnant rats via tail vein injection at 13-d and again on 14-d (term = 22–23 days). Saline-injected pregnant rats served as controls. Systolic blood pressure (SBP) of the pregnant rats were measured by a tail-cuff system (AD Instruments) at daily intervals after AT1-AA treatment. On day 21 of gestation, eight pregnant rats from each group were anesthetized and delivered by cesarean section. The numbers of live and dead fetal rats and the body weight of the live rats were record. Then, the fetal rats were used for collection of trunk blood after decapitation for the determination of AT1-AA titers in plasma. Two fetal rats heart were selected randomly from each pregnant rat for measurement of caspase-3 activity, and another two fetal rat heart were stained with hematoxylin-eosin (HE) staining and TUNEL staining and transmission electron microscopic examinations. The rest of pregnant rats (n=6 per group) continued until delivery. After birth, neonatal pups (3 males and 3 females) were killed by cervical dislocation at day 3, 7, 21 and 45, and the hearts were isolated and weighed to calculate the heart weight index. The hearts were also isolated from 45-day-old offspring (n=6~8 per group) for functional study. 

### AT1-AA Detection and Affinity Purification

AT1-AA extracted from the plasma of four patients with PE, that was diagnosed according to the criteria of the International Society for the Study of Hypertension in Pregnancy. The study was approved by the Ethics Committee of Shanghai Sixth People’s Hospital affiliated to Shanghai Jiao Tong University, and written informed consent forms were signed by all subjects. 

Serum antibody titer and purified antibody dilution were measured by using the ELISA method described previously [16,18]. Briefly, the peptides were coated (10 µg/ml in 100 mM Na_2_CO_3_) on 96-well plates. The wells were then saturated with PMT [phosphate-buffered saline supplemented with 5% (w/v) cow sera, 0.1% (V/V) Tween 20, and 0.01% (W/V) Thimerosal (Sigma, St Louis, USA)]. Fifty microliters of serial dilutions (doubling dilutions from 1:40 to 1:1280 in PMT) or purified antibody dilution were added to the saturated wells overnight at 4°C. An affinity-purified biotinylated goat anti-rat IgG was allowed to react for 1 h, followed by detection using streptavidinperoxidase (1 mg/ml) (Sigma), and substrates H_2_O_2_ (2.5 mM) and 2.29-azino-di (ethylbenzothiazoline) sulfonic acid (2 mM) (Sigma). Optical densities at 405 nm were measured after 30 min by a microplate reader (Molecular Devices, Sunnyvale, USA). AT1-AA were purified by MAb Trap Kit (Amersham, USA) according to the manufacturer’s instructions. Before use, the purified antibody was diluted with PBS to a antibody titer of greater than 1:640 by ELISA detected.

### HE and TUNEL staining

 Fetal hearts of 21-day gestational age were fixed in 10% formaldehyde and dehydrated with graded concentrations of alcohol for embedding in paraffin. The heart tissue was sliced into 5 μm sections for HE and TUNEL staining. AT1-AA-induced apoptosis in culture of FRMCs was also detected by TUNEL staining.

TUNEL assays were performed with the use of ApopTag Plus Fluorescein In Situ Apoptosis Detection Kits according to the manufacturer’s instructions (Chemicon, Temecula, CA, USA). Dewaxed and rehydrated specimens were incubated in proteinase K (40 mg/ml) for one hour at 37°C, and were then treated with 3% H_2_O_2_ in methanol for 30 minutes at room temperature to block endogenous peroxidase. After adding equilibration buffer for five minutes at room temperature, the terminal deoxynucleotidyl transferase enzyme was pipetted on to the sections and incubated at 37°C for two hours. The reaction was stopped by incubating the sections in stop buffer for 30 minutes at 37°C. Anti-digoxigenin peroxidase was added to the slides, followed by incubation for 30 minutes at 37°C. Slides were stained with diaminobenzine for 10 minutes and counterstained with haematoxylin and observation by bright field microscopy (Leica). A total of 500 cells were counted for each specimen. The apoptotic index was defined as follows: apoptotic index (%) = 100 ×apoptotic cells/total cells. We stratified tumor specimens according to TUNEL staining into (≤10% or >10% stained cells.

For ultrastructural cytology, another heart slice were fixed in 2.5% glutaraldehyde in 0.1 M sodium cacodylate, pH 7.25, 2% sucrose, and 2 mM calcium chloride at 4°C. Following postfixation with osmium tetroxide and dehydration through graded ethanol, the tissue was embedded in PolyBed resin (PolySciences). Semi-thin (0.5 mm) sections were stained with uranyl acetate and lead citrate for transmission electron microscopy.

### Perfused rat hearts subjected to ischemia-reperfusion

 45-day-old offspring were systemically heparinized (500 U, i.p.) and anesthetized with sodium pentobarbital (60 mg/kg, i.p.). Hearts were rapidly excised, connected via the aorta to Langendorff apparatus, and perfused in a retrograde manner at a constant pressure of 60 mm Hg with Kreb’s solution of the following composition (mmol/L): NaCl 118, KCl 4.7, CaCl_2_ 2.5, MgSO_4_ 1.2, KH_2_PO_4_ 1.2, NaHCO_3_ 25, glucose 11. The Kreb’s solution was bubbled continuously with a gas mixture of 95% O_2_ and 5% CO_2_ (pH 7.4), and the temperature was maintained at 37°C throughout the experiment. A latex balloon filled with Kreb’s solution was inserted into the LV to record of LV hemodynamic parameters by a digital acquisition and analysis system (PowerLab/4SP, ADInstruments, Pty. Ltd, Castle Hill, Australia). The indices of hemodynamic parameters including heart rate (HR), LV developed pressure (LVDP), LV end diastolic pressure (LVEDP), the maximal rates of pressure rise/fall (±dp/dt_max_) were recorded. After the baseline recording, the hearts were subjected to 20-min global ischemia by stopping the reperfusion, followed by 60-min reperfusion. 

### Measurement of myocardial infarct size

 At the end of reperfusion, the LV was collected and sliced into six or five 1-mm transverse sections. Except the outermost section, the other sections were incubated in triphenyltetrazolium chloride in sodium phosphate buffer at 37°C for 20 min, and immersed in formalin for 24 h to enhance contrast between the stained and unstained areas. Each section was then photographed (Kodak digital camera), and the areas of myocardial infarction (MI) in each section were analyzed by computerized planimetry (Image-Pro Plus). 

The outermost section was HE stained, and the cross-sectional area (CSA) of myocardial cells was determined using the method described by our group previously^16^. The size of myocardial cells in each group was evaluated by measuring the cross-sectional area of cells using the image analysis system (CIAS-1000, China Daheng Group, Inc., Beijing, China). The mean myocardial cell area was calculated by measuring 100 cells from the HE stained sections.

### Statistical analysis

 Data are presented as mean ± SD. Student’s t-test or one-way ANOVA followed by post hoc Student- Newman-Keuls test was used to determine the maternal and fetal characteristics. The non-parametric Kolmogorov-Smirnov test was used to compare the curves of vascular reactivity between the study and control groups. The median intensities were compared by Mood's Median Test. Results were considered statistically significant at P<0.05. 

## Results

### Maternal and fetal general characteristics

 Maternal and fetal characteristics are described in Table 1, showing that there was no significant difference in initial SBP and body weight of the pregnant rats. However, SBP increased significantly in AT1-AA group 7 days after antibody injection or 21 days of pregnancy as compared with the control group. There was no significant difference between the initial weight and final weight of the pregnant rats. Mortality was significantly high, and body weight, heart weight, body length and placental weight were significantly smaller in fetal rats delivered by cesarean section that received a prenatal exposure to AT1-AA. In addition, the titer of AT1-AA in amniotic fluid was low in AT1-AA group. 

**Table 1:** Maternal and fetal characteristics.

	Control group	AT1-AA group
Initial mother weight **^*a*^**	226.5 ± 15.7	228.4 ± 16.3
Mother weight at term (g, day 21 of pregnancy) **^*a*^**	390.6 ± 21.6	378.8 ± 19.6
Initial mother systolic BP (mmHg) **^*a*^**	102.3 ± 5.3	103.5 ± 5.7
Mother systolic BP at term (g, day 21 of pregnancy) **^*a*^**	106.4 ± 6.2	138.6 ± 8.7 *
Number of live fetuses Medians (min.-max.)	12 (10-14)	10 (9-11)
Number of stillbirth Medians (min.-max.)	0 (0-1)	2(1-4) **^*#*^**
Fetal body length **^*b*^**	3.54 ± 0.24	3.52 ±0.27
Fetal prenatal body weight (g, day 21 of pregnancy) **^*b*^**	4.85 ± 0.18	4.32 ± 0.15 *
Fetal prenatal heart weight (mg, day 21 of pregnancy)**^*b*^**	0.021 ± 0.004	0.019 ± 0.008 *
Heart weight index (mg/g × 100) **^*b*^**	0.432 ± 0.02	0.439 ± 0.03
Placental weight (g) **^*b*^**	0.63 ± 0.07	0.53 ± 0.06 *
Fetal plasma AT1-AA titre	<1:10	1:34.5 ± 12.7 *

Results are expressed as mean ± SD of control and AT1-AA groups. ***^a^*** n = 6 for both control and AT1-AA groups. ***^b^*** n = 71 for control group and 63 for AT1-AA group. *P<0.05, by ANOVA test; ***^#^***P<0.01 by Mood's median test.

**Figure 1:**
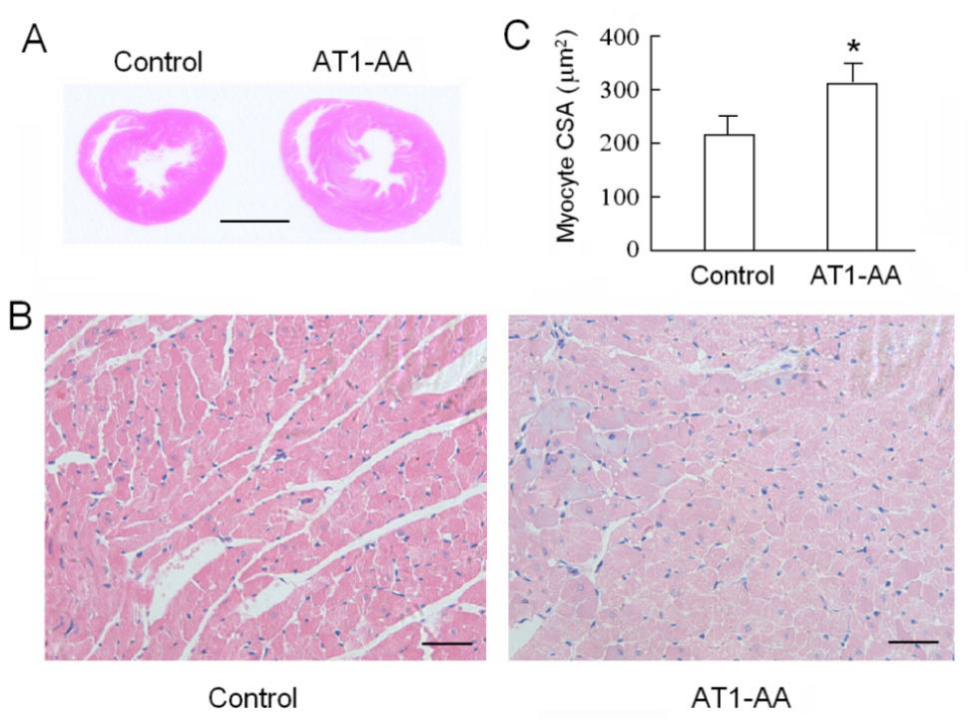
Effect of prenatal AT1-AA exposure on LV cross-sectional area of myocardial cells. Hearts were obtained from 45-day-old rats exposed to either saline or AT1-AA before birth from day 13 and 14 of gestational age. (A) Hematoxylin and eosin (H&E)-stained histological sections of hearts (bar, 3 mm); (B) Light-microscopic (400×) examination of cardiac tissue with H&E stained (bar, 100 µm); (C) The cross-sectional area (CSA) of myocardial cells in the left ventricular wall. Data are mean ± SD. *P<0.05, AT1-AA vs control, n=7–8.

As shown in Table 2, treatment with AT1-AA caused a significant decrease in body weight of 3- and 7-day neonates. However, the body weight of AT1-AA group was close to that of the control group 21 days after birth. The heart to body weight ratio was not significantly different between the two groups at all ages. The LV myocardial cell cross-sectional area of AT1-AA group was significantly increases as compared that of the control group at 45 days of age (P<0.05) (Figure 1). 

**Table 2:** The effect of prenatal AT1-AA exposure on postnatal body and heart weight.

Age	Group	n	BW(g)	HW(mg)	Heart weight index
Day 3	Control	6	8.85 ± 0.18	0.042 ± 0.007	0.534 ± 0.02
	AT1-AA	6	7.94 ± 0.16*	0.038 ± 0.007*	0.529 ± 0.02
Day 7	Control	6	16.5 ± 0.45	0.090 ± 0.009	0.487 ± 0.02
	AT1-AA	6	15.2 ± 0.52*	0.088 ± 0.008	0.493 ± 0.02
Day 21	Control	6	63.8 ± 1.39	0.306 ± 0.009	0.479 ± 0.02
	AT1-AA	6	65.7 ± 1.53	0.311 ± 0.008	0.473 ± 0.02
Day 45	Control	8	149.8 ± 6.37	0.645 ± 0.009	0.426 ± 0.02
	AT1-AA	7	156.2 ± 6.98	0.662 ± 0.008	0.427 ± 0.02

* Significance: P<0.05 AT1-AA vs. control rats (ANOVA).

### Histopathological Change in the Fetal Heart

**Figure 2:**
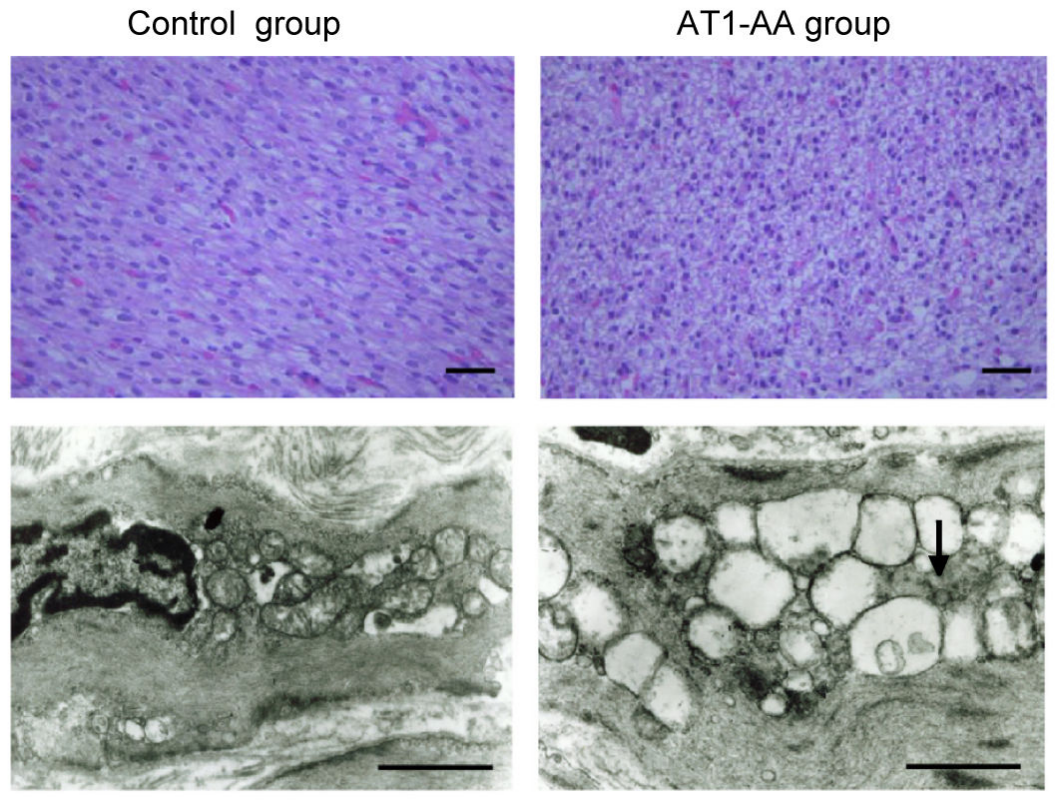
Changes in myocardial cells of fetal rats were observed with hematoxylin and eosin ( H&E) staining (A, B 400×; bar, 50 µm) and electron microscopy (C, D 10000×; bar, 1 µm). Hearts were obtained from fetal rats of 21 days gestational age exposed to either saline or AT1-AA before birth from day 13 and 14 of gestational age. A: The myocardial cells control were arranged and tightly stained uniform nuclei with round and clear nucleoli; B: the myocardial nuclei of AT1-AA group was significantly reduced; cells were deranged and swollen. C: myocardial cells arranged in neat rows with integrated mitochondria. D: mitochondrial volume was significantly more increased, and vacuoles enlarge.

 HE staining results of the fetal rats in AT1-AA group at 21-day pregnancy showed that the cardiac tissue underwent the following pathological changes: in the control group, the myocardial cells were oval or short spindled, arranged in neat dense rows with smaller intracellular spaces and tightly stained uniform nuclei with round and clear nucleoli, and no significantly abnormal change was seen in the cellular structure. In AT1-AA group, the myocardial nuclei number per each microscopic field was significantly reduced from control of 962.7 ± 67.4 to 728.6 ± 69.2 (P< 0.05); cells were deranged and swollen; the nuclei were deeply stained; and cytolymph and necrosis were observed in the myocardial tissue (Figure 2). Electron microscopic observation indicated that myocardial cells arranged in neat rows with integrated mitochondria in the control group fetal rats. In AT1-AA group, fetal rat myocardial cells wrinkled; the number of mitochondria was decreased; myofibrils were ruptured; sarcomeres blurred; and cellular nuclei were distorted (Figure 2). 

### Detection of cell apoptosis by TUNEL

**Figure 3:**
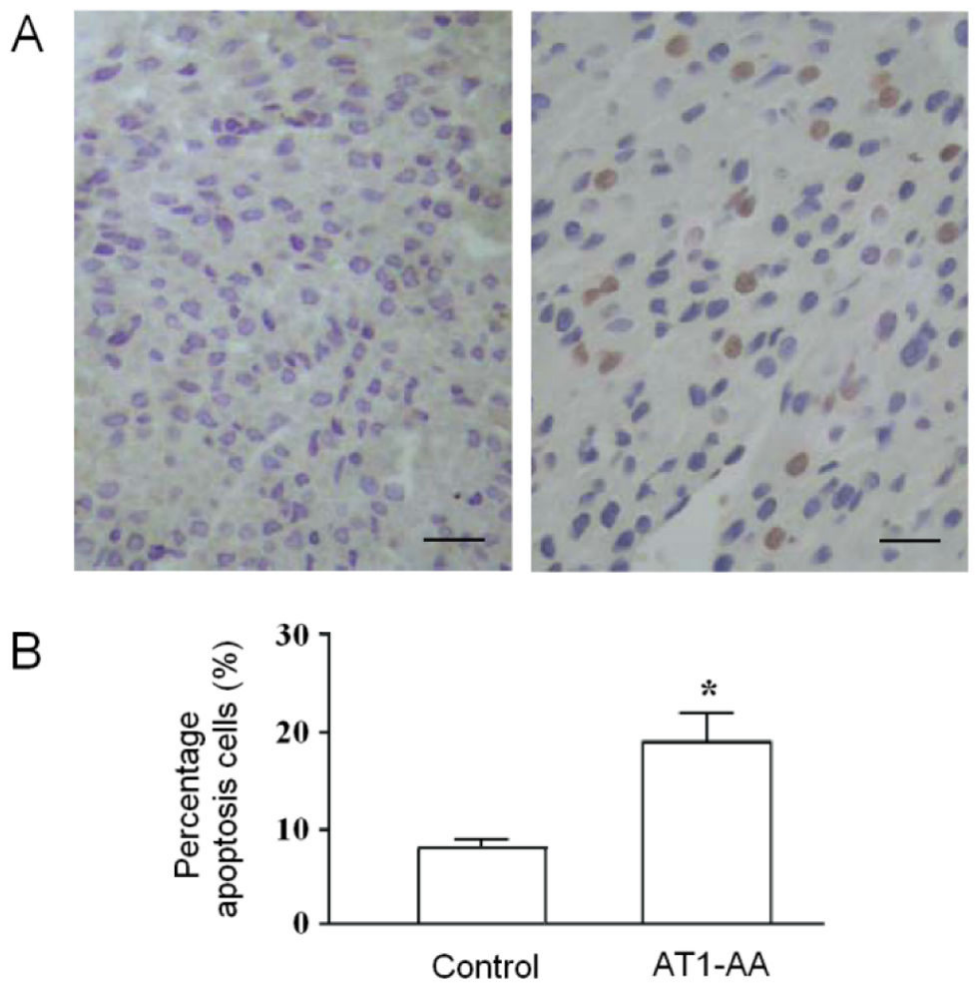
TUNEL assay of cardiac tissue obtained from fetal rats of 21 days gestational age exposed to either saline (A) or AT1-AA (B) before birth from day 13 and 14 of gestational age (200×, bar, 50 µm) . TUNEL-positive FRMCs showing shrunken cytoplasm, pycnotic nuclei, and/or apoptotic bodies increased. The percentage of TUNEL-positive myocardiac cells of each treatment is presented as the mean ± SD. C: Quantification of apoptotic cell death. *P<0.05, **P<0.01 vs. control.

Figure 3 shows TUNEL staining of the LV in AT1-AA and control groups. Myocardial cells of the control group arranged in neat rows and colored evenly, and some nuclei also showed signs of fragmentation. The result of image analysis showed that only a small amount of rat myocardial cell apoptosis was seen in the control group. However, in AT1-AA group, cell nuclei were stained yellow or brown, and some nuclei underwent karyopyknosis and karyolysis as compared with the control. The percentage of apoptotic myocardial cells in AT1-AA group was significantly higher than that in the control (P<0.01).

### LV function and post-ischemic recovery

**Figure 4:**
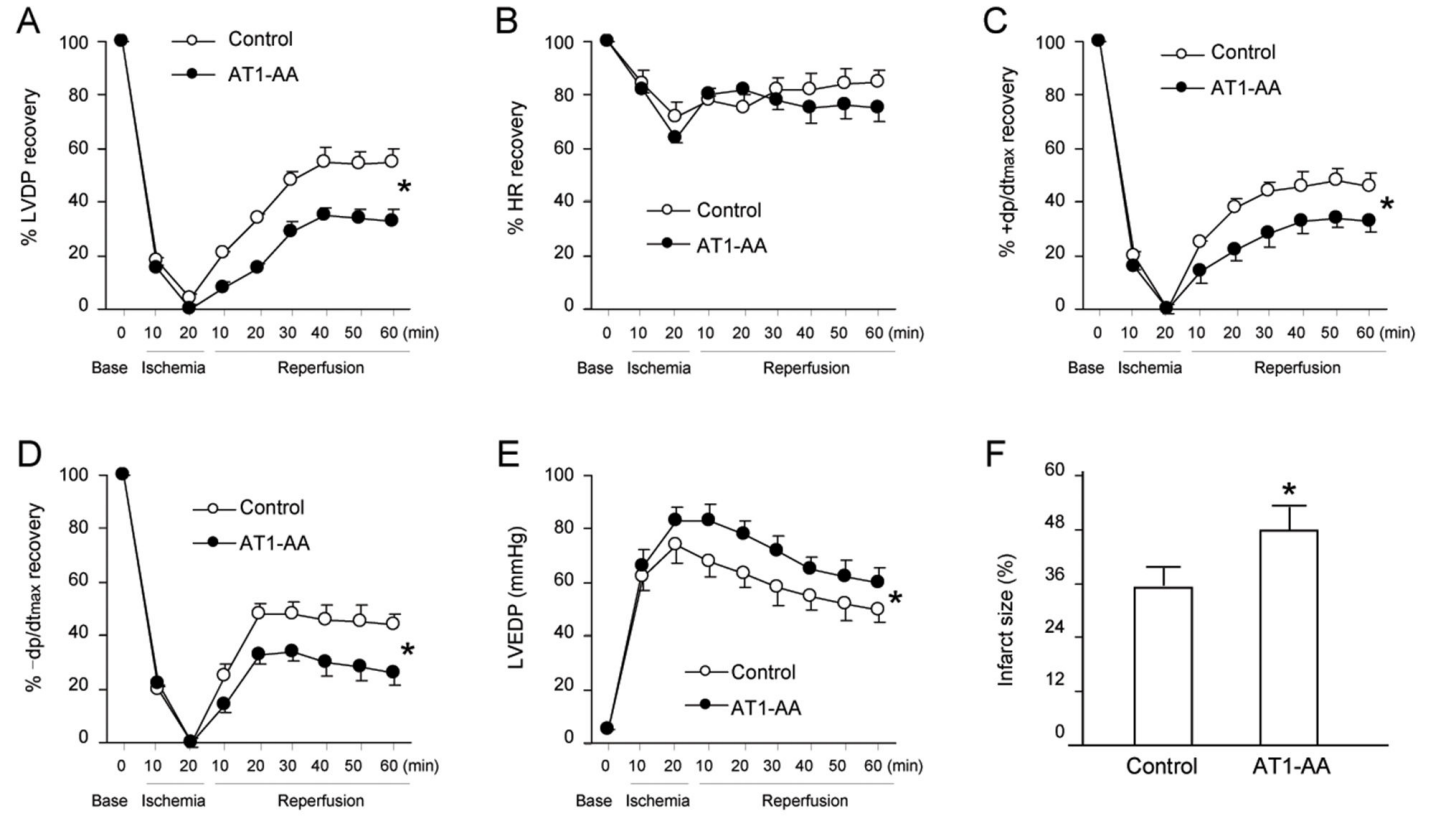
Effect of prenatal AT1-AA exposure on postischemic recovery of LV function (A-E) and myocardial infarction (F) in 45-day-old rats. Hearts obtained from 45-day-old rats exposed to either saline or AT1-AA (titer 1:640 0.1ml/kg) before birth at day 13 and 14 of gestational age were subjected to 20-min ischemia and 60-min reperfusion in the Langendorff preparation. LVDP, left ventricular developed pressure; HR: heart rate; ±dp/dt_max_; the maximal rates of pressure rise/fall; LVEDP: left ventricular end diastolic pressure. LV were collected at the end of reperfusion, and myocardial infarct size was determined with 1% TTC staining and expressed as a percentage of the total LV weight. Data were analyzed by two-way ANOVA with ischemia–reperfusion as one factor and AT1-AA treatment as the other. *P<0.05 compared with control for the entire curve. n=6-8 per group.

 Using the Langendorff preparation, LV function was assessed in isolated hearts from 45-day-old offspring that were exposed to either saline or AT1-AA before birth. The experimental results showed that there was no significant difference in LV functional parameters at baseline levels between males and females in the same group, and also no difference (except for HR) between control group and AT1-AA treated group. Figure 4 shows the effect of 20-min ischemia followed by 60-min reperfusion on LV function in the two groups. Cardiac contraction completely stopped or slowed down during 20-min ischemia, but gradually resumed when perfusion was restored. Compared with the control group, post-ischemic recovery of the functional parameters in AT1-AA-treated animals was delayed significantly, but there was no significant difference in post-ischemic recovery of HR between the two groups. The infarct size of LV at the end of 60 min reperfusion after 20 min ischaemia is shown in Figure 4. Prenatal AT1-AA treatment significantly increased infarct size in the heart of offspring (Figure 4F).

## Discussion

PE is a condition severely endangering both mothers and fetuses. It is the main cause of maternal death, premature birth and fetal death. The main pathological change of PE is vascular spasm and water-salt retention, which reduces the renal filtration function and increases BP, resulting in proteinuria, edema and fetal developmental retardation. As the pathogenesis of human PE remains unclear, PE models established by injecting endotoxins [19], or NO synthase inhibitors [20], into fetal rats are ineffective. Ample evidence has shown that AT1-AA participates in the pathogenesis of PE. Zhou et al. succeeded in established a PE model by injecting plasma from women with PE containing AT1-AA IgG into fetal rats [21]. We believe that the preeclampisa model established by this method can more truly reflect the condition of human PE, and therefore we used this model in the present study to observe the effect of AT1-AA on the heart structure and function of fetal rats. According to the dosage and expected experimental result reported by Zhou et al [21], we selected 0.1ml/Kg.body weight AT1-AA of 1:640 titer in this study and found that AT1-AA at this dosage caused BP to increase continuously and proteinuria without causing death of the rats.

PE is the main cause contributing to fetal growth restriction and placental distress, resulting in premature birth and death, or even increasing the risk of after-birth metabolic disorders such as hypertension, coronary heart disease and lipid abnormalities [9,22]. Irani et al. [17] successfully established a rat PE model by injecting AT1-AA extracted from the plasma of patients with into fetal rats, which also induced developmental retardation of the fetal rats in the uterus. Studies also showed that pathogenic AT1-AA could gain access to the placenta, affecting the placental development and inducing apoptosis of placental trophoblastic cells, or gain access to the fetal circulation via the umbilical cord, affecting the normal growth and maturation of the fetal liver and kidney. In the present study, we found for the first time that AT1-AA could not only induce PE in pregnant rats but gain access to the fetal circulation via the placenta to induce apoptosis of myocardial cells in fetal rats. Apoptosis of myocardial cells during the fetal phase may be the cause for myocardial remodeling soon after birth, and the cause of increased susceptibility of the heart to IRI

It was found in our study that weight of the placenta and the body weight of the neonatal rats in AT1-AA group were decreased significantly as compared with the animals in the control group. Irani et al. [17] also found in their earlier study that the body weight of neonates born to AT1-AA-induced preeclamptic mice was also significantly lower than that of the control group. The result of our study showed that the body weight of the neonatal rats in AT1-AA group decreased by about 11% as compared with that of the control group, which is consistent with the finding that the body weight of neonates born to women with PE decreased by about 10%. We believe that the effect of AT1-AA on vascular contraction can cause fetal hypoxia, which is one of the factors contributing to fetal growth retardation and weight reduction [13,14].

Cell apoptosis is a gene-mediated process of spontaneous cell death. In the development and growth of an organism, apoptosis gets rid of physiologically unwanted cells, thus normalizing the functions of various tissues and organs of an individual. Apoptosis occurs in the sinus node, atrioventricular node and conductive tissues during embryonic development and in the early stage of development after birth. Excessive, insufficient or delayed apoptosis of these cells may cause dysrhythmia [23]. Caspase is the key enzyme causing cell apoptosis. Under normal conditions, it exists in normal cells in the form of a non-active proenzyme. Caspase-3 is the “core” proteinase in apoptosis protease cascade reaction, acting as the “co-central processing unit” in the apoptosis pathway. It can enzymolyse cellular structural protein, cut off the link between apoptotic cells and surrounding cells, close DNA relication and repair, degrade DNA, and finally decompose and envelop cells to form apoptotic bodies. Our previous study showed that AT1-AA could induce apoptosis of myocardial cells of neonatal rats in a dose-dependent manner, and AT1-AA could enhance caspase-3 activity in a dose- and time-dependent manner and cause apoptosis of neonatal rats myocardial cells [15]. It was found that the TNF-α concentration in the medium treated with AT1-AA began increasing 2 h after culture and reached the peak at 6 h (8 times as high as the control group) [15]. The apoptosis-inducing effect of AT1-AA was attenuated significantly after the use of AT1R and TNF-α inhibitors, indicating that the apoptotic effect of AT1-AA on myocardial cells is associated with AT1R activation and the production of TNF-α. It was found in our study that the body weight of the fetal rats in AT1-AA group was significantly lower than that of the control group, and that the extent and intensity of caspase-3 expression were also significantly higher than those of the control group. Histological study of the hearts showed that the nuclei of most myocardial cells of the control group were regularly arranged, and part of the intranulcear karyotin was not evenly distributed, presenting characteristics of apoptotic nuclei, indicating that apoptosis of myocardial cells existed during the normal development and growth of the fetal rats. In AT1-AA group, the size of myocardial nuclei of the fetal rats was small and irregular, presenting with aggregation of karyotin and other apoptotic characteristics, indicating that entry of AT1-AA from the pregnant rats to the fetal rats could significantly increase apoptosis of myocardial cell of the fetal rats. 

As myocardial cells are highly differentiated and do not proliferate after birth any longer, myocardial cells lost during apoptosis will permanently reduce the basic unit of myocardial contraction. It was found in our study that the heart weight of fetal rats in AT1-AA group was significantly lower than that of the control group at day 3 after birth (P<0.05), but there was no significant difference in heart weight and heart weight index between the two groups at day 7 after birth. The cross-sectional area of myocardial cells of the offspring rats in AT1-AA group was significantly larger than that in the control group, indicating that the offspring rats in AT1-AA group compensated for the loss of myocardial cells during apoptosis by increasing the area of the myocardial cells during heart remodeling after birth. Some studies [24-26] reported that energy metabolism pathways underwent significant changes during myocardial hypertrophy, presenting as decreased fatty acid oxidation, increased glucose utilization, and decreased expression of mRNA and protein that regulate fatty acid oxidation. This metabolic pattern seen during the embryonic period reduces the tolerance of myocardial cells to myocardial ischemia and functional recovery after ischemia [1]. It was found in our study that the cardiac function at rest was not significantly affected in AT1-AA-treated rat offspring but recovery of the cardiac function from IRI was poorer and the area of myocardial infarction was larger, which are probably related to apoptosis- induced myocardial hypertrophy due to AT1-AA treatment during the fetal stage. 

To the best of our knowledge, this is the first study demonstrating that offspring born to AT1-AA-treated pregnant rats had increased susceptibility to IRI, increased area of myocardial infarction, and decreased ability to recover LV function from ischemia. AT1-AA-induced apoptosis of fetal myocardial infarction may cause lasting and life-long influence on cardiac function, and may be the main factor contributing to the increased incidence of CVD after birth.
